# Ca^2+^ Regulation of TRP Ion Channels

**DOI:** 10.3390/ijms19041256

**Published:** 2018-04-23

**Authors:** Raquibul Hasan, Xuming Zhang

**Affiliations:** 1 Department of Pharmaceutical Sciences, College of Pharmacy Mercer University, Atlanta, GA 30341, USA; shasan6@uthsc.edu; 2 School of Life and Health Sciences, Aston University, Aston Triangle, Birmingham B4 7ET, UK

**Keywords:** TRP ion channels, Ca^2+^ signaling, calmodulin, phosphatidylinositol-4,5-biphosphate (PIP_2_)

## Abstract

Ca^2+^ signaling influences nearly every aspect of cellular life. Transient receptor potential (TRP) ion channels have emerged as cellular sensors for thermal, chemical and mechanical stimuli and are major contributors to Ca^2+^ signaling, playing an important role in diverse physiological and pathological processes. Notably, TRP ion channels are also one of the major downstream targets of Ca^2+^ signaling initiated either from TRP channels themselves or from various other sources, such as G-protein coupled receptors, giving rise to feedback regulation. TRP channels therefore function like integrators of Ca^2+^ signaling. A growing body of research has demonstrated different modes of Ca^2+^-dependent regulation of TRP ion channels and the underlying mechanisms. However, the precise actions of Ca^2+^ in the modulation of TRP ion channels remain elusive. Advances in Ca^2+^ regulation of TRP channels are critical to our understanding of the diversified functions of TRP channels and complex Ca^2+^ signaling.

## 1. Introduction

Transient receptor potential (TRP) ion channels are a large family of cation channels consisting of six mammalian subfamilies: TRPV, TRPM, TRPA, TRPC, TRPP and TRPML [1]. They are activated by a vast array of stimuli in the environment, ranging from temperature, natural chemicals and toxins, to mechanical stimuli. They also respond to endogenous agents and messengers produced during tissue injury and inflammation [2]. TRP channels therefore function as molecular sensors responsible for detecting the external world and internal milieu, and have been implicated in diverse physiological processes and diseases, such as temperature sensation, vision, taste, pain, itch and cardiovascular diseases [1,2,3,4].

The TRPV subfamily consists of six members. Of them, TRPV1–4 are mainly expressed in the sensory ganglia and skin responsible for thermo-sensation, pain and itch, whilst TRPV5 and TRPV6 are primarily expressed in the kidney and gastrointestinal tract and play a major role in Ca^2+^ absorption and homeostasis [3,5,6]. The TRPM subfamily is the largest, consisting of eight members broadly expressed in a variety of cells and tissues, such as sensory ganglia, pancreatic beta cells, immune cells, the tongue, heart and kidney, and are critical for sensory physiology (e.g., heat (TRPM3), cold (TRPM8), taste (TRPM5) and light (TRPM1) sensing and detection) [5], insulin release (TRPM2, TRPM4 and TRPM5) [7,8], Mg^2+^ homeostasis (TRPM6 and TRPM7), ischemic injury and inflammatory responses (TRPM2, TRPM4) [9]. TRPA1 is the sole member of the TRPA subfamily, implicated in pain, itch and inflammation [10]. TRPC is another big TRP subfamily that includes seven subtypes widely expressed in the central nervous system and various other tissues participating in diverse conditions such as neuron development and epilepsy [5,11]. TRPP channels mainly cause polycystic kidney disease due to mutations in the channel genes. In contrast to other TRP subfamilies largely expressed on cell membranes, TRPML channels are mainly localized in endosomes and lysosomes critical for lysosome storage disorder, vesicle trafficking and ion homeostasis [12]. These TRP ion channels are modulated by various mechanisms, most commonly by membrane voltage, phosphoinositides, phosphorylation and G-protein coupled receptor signaling [5].

Notably, most TRP ion channels are permeable to Ca^2+^ except TRPM4 and TRPM5 [2]. Activation of TRP channels therefore also serves as an important Ca^2+^ entry pathway contributing to fluctuations in intracellular Ca^2+^ ([Ca^2+^]_i_) and subsequent Ca^2+^ signaling. Ca^2+^ is a ubiquitous second messenger participating in numerous signal transduction pathways involved in biological processes as diverse as secretion, gene transcription and cell death [13]. Furthermore, similar to other cations, Ca^2+^ ions contribute to the electrochemical gradient in excitable cells and are critical to cellular excitability. Maintenance of Ca^2+^ homeostasis is therefore crucial for the functions of both excitable and non-excitable cells. Interestingly, many TRP ion channels have evolved sophisticated auto-regulatory mechanisms allowing them to self-regulate Ca^2+^ entry for the tight control of Ca^2+^ fluctuations and downstream Ca^2+^ signaling. Understanding the Ca^2+^-dependent regulation of TRP ion channels is therefore not only essential for elucidating TRP channel mediated physiology and diseases, but also critical for understanding the Ca^2+^ signaling network.

Calmodulin (CaM), a founding member of Ca^2+^-binding proteins, is a fascinating Ca^2+^ signaling regulator. Firstly, CaM exhibits high sensitivity to Ca^2+^ conferred by the four EF-hand domains, a well-characterized Ca^2+^-binding motif. These EF-hand domains form two globular lobes (N- and C-lobe), with each lobe containing two EF-hand domains but with differential Ca^2+^-binding affinity (*K*_d_ for N-lobe, 10^−6^ M; *K*_d_ for C-lobe, 10^−7^ M) [14]. Two lobes of CaM act as two independent Ca^2+^ sensors for decoding different Ca^2+^ signals and are the mechanistic origins of the lobe-specific regulation of P/Q-type Ca^2+^ channels and TMEM16A Cl^−^ channels by different Ca^2+^ levels [15,16]. Secondly, CaM assumes various conformations endowed by two independent lobes and a long flexible linker connecting the two lobes, allowing CaM to bind to and regulate a myriad of target proteins [17], including ion channels, such as small-conductance K^+^ (SK) channels [18,19], KCNQ channels, cyclic nucleotide-gated channels and voltage-gated Na^+^ channels [20,21]. These unique properties of CaM suggest a broad role for CaM in the Ca^2+^-dependent regulation of TRP ion channels.

Indeed, a growing body of research has demonstrated the wide involvement of CaM in the Ca^2+^ regulation of TRP ion channels, including the TRPV, TRPM, TRPA1 and TRPC subfamily channels. These studies have substantially expanded the function of CaM and advanced our understanding of TRP channel modulation. Apart from CaM, Ca^2+^ modulates TRP channels by direct binding to TRP ion channels and by activating Ca^2+^-dependent enzymes. Here, we review the recent progress in the Ca^2+^ regulation of TRP ion channels, focusing on TRPV, TRPA, TRPM and TRPP channels. The Ca^2+^ regulation of TRPC channels is explained in the review by Zhu MX [22].

## 2. TRPV1

Capsaicin has traditionally been used as an analgesic for relieving pain. However, the analgesic mechanisms of capsaicin are poorly understood. The discovery of TRPV1 as a capsaicin receptor and its role in inflammatory pain suggested TRPV1 as the target of the analgesic actions of capsaicin [23,24,25]. It was soon found that persistent TRPV1 activation by capsaicin evokes channel desensitization and tachyphylaxis, a process that is dependent on Ca^2+^ and thought to underlie the analgesic effect of capsaicin [25,26]. However, how Ca^2+^ induces TRPV1 desensitization remains unclear.

Several studies have implicated a role for CaM in Ca^2+^-dependent desensitization of TRPV1, though there is a discrepancy regarding the precise regions in TRPV1 targeted by CaM [27,28,29]. A 35 amino acid peptide region in the distal C-terminus of TRPV1 was initially proposed as the CaM binding domain (CaMBD), because deletion of this region abolished CaM binding and blunted TRPV1 desensitization [27]. Consistently, this region was found to bind to two lobes of CaM with high affinity (*K*_d_ = 5.4 × 10^−8^ M) in an antiparallel orientation through hydrophobic and electrostatic interactions [30,31]. Soon after, a second CaMBD was identified overlapping with the first ankyrin repeat domain (ARD) in the N-terminus of TRPV1 (N-CaMBD) [28,29]. Interestingly, this region coincidently binds to ATP [29], which is known to enhance TRPV1 activity and prevent TRPV1 desensitization [32,33]. It was therefore proposed that activated Ca^2+^/CaM as a result of TRPV1 opening and Ca^2+^ influx binds to the ARD and displaces ATP, leading to channel inactivation and desensitization [29]. In further support of this idea, mutating the ATP binding sites on the ARD abolished Ca^2+^/CaM binding to the ARD and prevented Ca^2+^-dependent TRPV1 desensitization [29,30]. Although C-CaMBD is a high affinity Ca^2+^/CaM binding site, N-CaMBD appears to play a more important role in TRPV1 desensitization [30].

**Figure 1:**
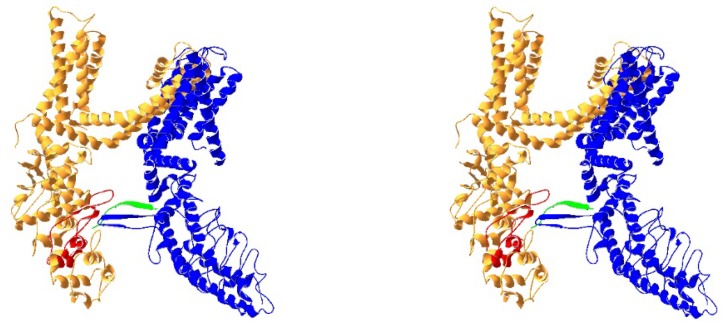
Ribbon diagram of TRPV1 structure (PDB, 3J5P) showing proximity of the first ARD (red) in the N-terminus of TRPV1 to the distal C-terminus (green) from an adjacent TRPV1 subunit (stereo view in parallel).

How do these two CaMBDs act in concert to mediate TRPV1 desensitization? An interesting possibility is that Ca^2+^/CaM binds to both N- and C-CaMBD through two independent lobes forming a tertiary complex, resulting in cross-linking or dimerization of two adjacent TRPV1 subunits, as seen in small conductance Ca^2+^-activated K^+^ (SK) channels [19]. This possibility is also supported by the resolved TRPV1 structure showing that the distal C-terminus of TRPV1 was in contact with the ARD from an adjacent TRPV1 subunit (Figure 1) [34]. However, this possibility was not demonstrated in size exclusion chromatography (SEC) through analysis of the binding of two TRPV1 CaMBD fragments [30]. Structural determination of TRPV1 in complex with Ca^2+^/CaM may be a better approach to resolve this possibility in the future.

In addition to a direct effect on TRPV1, Ca^2+^/CaM also activates a number of downstream kinases and phosphatases, such as protein phosphatase 2B (PP2B, calcineurin) [17]. A role for PP2B in the Ca^2+^-dependent desensitization of TRPV1 was demonstrated in experiments showing that cyclophilin, a specific PP2B inhibitor, prevents TRPV1 desensitization [35,36]. Interestingly, we further found that PP2B mediated TRPV1 desensitization depends on AKAP79/150, a scaffold protein anchoring PKA, PKC and PP2B in close proximity to TRPV1 through binding to the C-terminus of TRPV1 [37], because the Ca^2+^ desensitization of TRPV1 was reduced by either downregulating AKAP79, by specific deletion of the PP2B binding region on AKAP79, or by a peptide disrupting TRPV1–AKAP79 interaction [37]. However, this finding was not reproduced using calcium imaging [38]. It should be noted that the fluorescent dye used in calcium imaging binds to Ca^2+^, therefore buffering Ca^2+^-dependent downstream processes. Furthermore, calcium imaging is an indirect measurement of TRPV1 activity. In contrast, we used whole-cell patch clamping to directly record TRPV1 currents and selected cells with similar peak currents for experiments, as TRPV1 desensitization critically depends on the initial level of Ca^2+^ influx. These differences may account for the negative finding by Por et al. [38].

Not surprisingly, Ca^2+^-dependent desensitization of TRPV1 is subject to modulation by PKA and PKC, which are known to sensitize TRPV1 by enhancing TRPV1 phosphorylation [39,40,41]. Activation of either PKA or PKC reversed TRPV1 desensitization [36,39,42,43,44]. These data suggest that PP2B promotes TRPV1 desensitization by dephosphorylation of TRPV1, counteracting channel phosphorylation by protein kinases. However, it remains unknown whether PP2B dephosphorylates the same phosphorylation sites targeted by PKA and PKC.

In addition to Ca^2+^/CaM-dependent regulation of TRPV1 desensitization, considerable evidence also supports the involvement of membrane phosphatidylinositol-4,5-biphosphate (PIP_2_). Firstly, PIP_2_ has been shown to activate TRPV1 in excised patches [29,45,46,47]; secondly, Ca^2+^ influx during TRPV1 activation substantially decreases PIP_2_ [48]; thirdly, Ca^2+^-dependent desensitization of TRPV1 can be prevented either by inhibition of PIP_2_ degradation with a phospholipase C (PLC) inhibitor or by supplementing PIP_2_ through the patch pipette [29,46]; and lastly, deletion of a Ca^2+^-sensitive PLC significantly has been shown to reduce TRPV1 desensitization [48]. These findings support a critical role for Ca^2+^-induced hydrolysis of PIP_2_ in TRPV1 desensitization.

**Figure 2:**
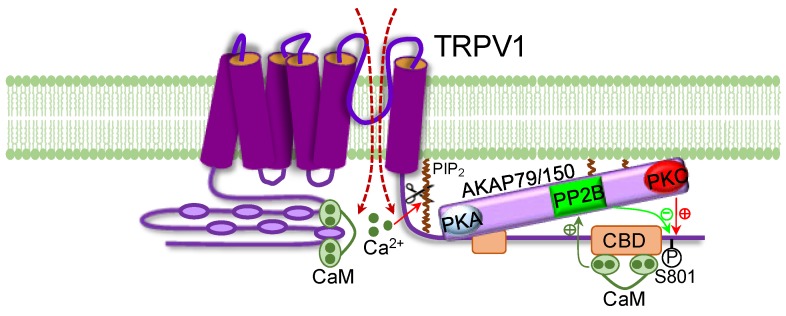
Schematic diagram depicting Ca^2+^-dependent modulation of TRPV1 desensitization. Influxed Ca^2+^ binds to CaM. Ca^2+^-loaded CaM then binds to the ARD in the N-terminus of TRPV1, displacing the binding of ATP, leading to channel inhibition. Ca^2+^/CaM also binds to the CaM-binding domain (CBD) in the distal C-terminus and activates PP2B anchored on AKAP79/150 that binds to the proximal C-terminus of TRPV1. Activated PP2B removes and antagonizes phosphorylation of TRPV1 (S801) by PKC and PKA that are also anchored on AKAP79/150. Meanwhile, Ca^2+^ induces hydrolysis of PIP_2_, further promoting channel desensitization.

Based on these findings, we synthesize a model of Ca^2+^-induced desensitization of TRPV1. Upon TRPV1 opening and Ca^2+^ influx, Ca^2+^/CaM becomes activated and binds to both N- and C-CaMBD, inducing structural changes in TRPV1, leading to channel inactivation. The high affinity binding of Ca^2+^/CaM to C-CaMBD also activates nearby PP2B anchored on AKAP79/150 that binds to a nearby upstream region of C-CaMBD. PP2B then dephosphorylates TRPV1, counteracting channel phosphorylation by anchored PKA and PKC on AKAP79/150, promoting TRPV1 desensitization (Figure 2). Meanwhile, Ca^2+^ activates Ca^2+^-sensitive PLC, resulting in the cleavage of membrane PIP_2_, further strengthening channel desensitization.

## 3. TRPV2

TRPV2 was initially characterized as a sensor for noxious heat, similar to TRPV1 [49]. However, mice lacking TRPV2 were shown to exhibit normal heat sensation [50], arguing against this initial hypothesis. Later on, it was found that TRPV2 is critical for macrophage phagocytosis and innate immunity [51], and is also involved in cancer migration and insulin secretion [52].

Like TRPV1, TRPV2 also exhibits Ca^2+^-dependent desensitization [53]. However, unlike TRPV1, the ARD in the N-terminus of TRPV2 does not bind to CaM or ATP [53,54]. Although an equivalent CaMBD has been identified in the proximal C-terminal region of TRPV2 [53,55], Ca^2+^/CaM binding did not play a role in Ca^2+^-induced TRPV2 desensitization [53]. Instead, desensitization of TRPV2 was found mediated by a concomitant reduction in the membrane PIP_2_ as a result of Ca^2+^ entry through the channel, because Ca^2+^-dependent TRPV2 desensitization was reduced by dialyzing cells with soluble diC8-PIP_2_ through the patch pipette [53]. Presumably, increased Ca^2+^ through TRPV2 preferentially activates a Ca^2+^-sensitive PLC, leading to cleavage of PIP_2_. This is a possibility remaining to be investigated. The functional significance of TRPV2 desensitization is unclear, but it likely protects cells from the toxic effects of excessive Ca^2+^.

## 4. TRPV3

TRPV3 is also a thermosensitive ion channel activated by temperature ranges above 30–33 °C [56]. However, the role of TRPV3 in thermosensation in animals has not been consistently demonstrated [57,58,59]. An emerging theme is that TRPV3 plays a prominent role in skin physiology and diseases such as dermatitis [6,56].

A key feature of TRPV3 is that repetitive TRPV3 activation sensitizes its own responses, a process critically dependent on Ca^2+^ [60]. This Ca^2+^-dependent sensitization is in sharp contrast to Ca^2+^-induced desensitization of TRPV1 and TRPV2. Interestingly, sensitization of TRPV3 by Ca^2+^ is mediated by a conserved Ca^2+^/CaM and ATP binding site in the ARD in the N-terminus, analogous to that in TRPV1. In contrast to sensitization of TRPV1 by ATP, TRPV3 has been shown to be inhibited by ATP and also by Ca^2+^/CaM binding [54,60], which acts by shifting channel voltage dependence towards positive potentials [60]. This has been demonstrated by several approaches, including buffering increases of [Ca^2+^]_i_, pharmacological inhibition of CaM, depletion of CaM with anti-CaM monoclonal antibody, and disrupting CaM binding through mutation, all of which potentiated TRPV3 currents, and reduced or abolished channel sensitization and voltage dependence [54,60]. It was therefore proposed that repetitive TRPV3 activation reduces the binding affinity of TRPV3 to Ca^2+^/CaM, leading to channel disinhibition and consequently channel sensitization. However, whether there is an actual reduction in Ca^2+^/CaM-TRPV3 binding during channel activation remains to be investigated.

A second mechanism for Ca^2+^-dependent modulation of TRPV3 arises from a direct Ca^2+^ block at a negatively charged residue, Asp641, in the pore region of the channel. Mutating this residue eliminated high affinity inhibition by Ca^2+^ (<10 μM), while sparing the effect of low affinity inhibition by Ca^2+^, which is mediated by Ca^2+^/CaM as described above [60]. TRPV3 therefore employs two distinct mechanisms to mediate channel sensitization by Ca^2+^, which is believed to underlie enhanced skin sensitivity to allergens and skin sensitizers [61].

## 5. TRPV4

TRPV4 is another member of thermosensitive TRP channels activated by low osmolarity and warm temperatures (≥27 °C) [3], and has been implicated in mechanical transduction, pain and itch [62]. Mutations in TRPV4 cause many genetic disorders, such as Charcot–Marie–Tooth disease and spinal muscular atrophy, due to dysregulated activation of TRPV4 [63].

Similar to other thermo-TRPs, Ca^2+^ also regulates TRPV4, but exerts a dual effect, initially promoting channel activation followed by channel inactivation [64]. Potentiation of TRPV4 can be induced by Ca^2+^ released from intracellular stores or by extracellular Ca^2+^ influx through the channel pore [64,65], suggesting that Ca^2+^ acts on intracellular channel sites. Consistently, two different CaMBDs were identified by two independent groups: one in the ARD in the N-terminus (N-CaMBD) (P132-I383) that also binds to ATP, analogous to that found in TRPV1 and TRPV3 [54]; and another in the C-terminus of TRPV4 (C-CaMBD, H812-E831) [65]. Disrupting CaM binding to C-CaMBD through mutation abolished Ca^2+^-induced potentiation of TRPV4 and significantly slowed down channel activation rate [65]. Mechanistically, it was initially proposed that Ca^2+^/CaM binding displaces and releases an inhibitory interdomain interaction with C-CaMBD from the N-terminus of TRPV4, similar to CaM-dependent activation of CaMKII [17]. This idea was supported by the finding that disrupting the interaction between the N-terminus and C-CaMBD enhanced TRPV4 currents, as did Ca^2+^/CaM, and also abolished Ca^2+^-induced channel potentiation [66]. However, a different autoinhibitory domain in the C-terminus (795–804), immediately upstream of C-CaMBD, was demonstrated to be critical. Firstly, deleting this domain resulted in constitutive channel activity and eliminated Ca^2+^/CaM modulation; and secondly, two gain-of-function TRPV4 mutants, E797K and P799L, underlying heritable skeletal dysplasia are also within the proposed autoinhibitory domain and exhibited reduced modulation by Ca^2+^/CaM [67]. Furthermore, C-CaMBD also mediates sensitization of TRPV4 by inositol triphosphate (IP_3_), as deletion of C-CaMBD abolished IP_3_ binding and also prevented sensitization of TRPV4 by IP_3_ [68]. C-CaMBD may therefore be a converging point through which Ca^2+^ and other modulators regulate TRPV4 activation.

Although the role of C-CaMBD in Ca^2+^-dependent regulation of TRPV4 is clear, the role of N-CaMBD in this process remains elusive. Notably, N-CaMBD not only binds to ATP and CaM, but also directly binds to PIP_2_ [69]. Functionally, TRPV4 is sensitized by ATP [54], but inhibited by PIP_2_ [69]. The net effect of Ca^2+^/CaM binding to N-CaMBD is therefore uncertain. Ca^2+^/CaM could prevent ATP and/or PIP_2_ from binding to N-CaMBD, causing either channel inactivation, as seen in TRPV1 [29], or channel potentiation. Interestingly, N-CaMBD (P132-I383) also overlaps with the initially proposed inhibitory domain (S117-K136) in the N-terminus [66]. Therefore, it is tempting to speculate that Ca^2+^/CaM binds to both N- and C-CaMBD through two different lobes forming a tertiary complex coupling Ca^2+^ signals to channel activation, as demonstrated for SK channels [18], though this possibility has not been supported by protein binding assay [66]. In general, Ca^2+^-induced TRPV4 potentiation has been well-studied. However, study on Ca^2+^-induced TRPV4 inactivation is still lacking. Whether it is mediated by Ca^2+^/CaM as suggested above remains an open question.

## 6. TRPV5 and TRPV6

TRPV5 is mainly expressed in epithelial cells in the kidney, playing a major role in renal Ca^2+^ reabsorption, whilst TRPV6 is expressed in intestine epithelial cells responsible for Ca^2+^ absorption [70]. Therefore, both TRPV5 and TRPV6 are critical for systemic Ca^2+^ homeostasis [70]. Consistent with their functional role, TRPV5 and TRPV6 are highly selective for Ca^2+^, acting as specialized epithelial Ca^2+^ channels and the gatekeepers of transcellular Ca^2+^ transport. This is in contrast to other TRP channels that are nonselective to cations.

The high Ca^2+^ selectivity is determined by a single aspartate residue (TRPV5-D542, TRPV6-D541) in the pore forming region, as mutation of these residues abolishes Ca^2+^ permeation of TRPV5 and TRPV6 [70,71]. Once opened, Ca^2+^ entry rapidly triggers inactivation of TRPV5 and TRPV6 with TRPV6 undergoing a much faster inactivation rate than TRPV5, providing a Ca^2+^-dependent feedback mechanism for preventing excessive Ca^2+^ influx [72].

Similar to other TRPV channels, CaM is responsible for Ca^2+^-induced inactivation of TRPV5 and TRPV6. For TRPV5, a distal C-terminal CaMBD (696–729) is crucial [73,74]. Mutating two residues, W702 and R706, in this region has been shown to diminish Ca^2+^-dependent channel inactivation [74]. An equivalent distal C-terminal CaMBD (695–727) was also found in TRPV6. Deleting this region not only abolished Ca^2+^-dependent interaction between TRPV6 and CaM, but also significantly blunted Ca^2+^-elicited channel inactivation [75,76]. Furthermore, overexpressed CaM, but not CaM_1234_, in which the four Ca^2+^-binding sites are mutated and therefore Ca^2+^-insensitive, exhibited a Ca^2+^-dependent interaction with TRPV6 and correspondingly reduced TRPV6 currents [75]. These data support a dynamic rather than a constitutive interaction of CaM with TRPV6 [75,77]. CaM binding regions were also identified in other regions of TRPV5 and TRPV6, including the N-terminus, transmembrane region and other parts of C-terminus [55,77,78]. However, Ca^2+^-dependent interaction with CaM was barely observed for some of the postulated CaMBDs in live cells [75], and importantly, no functional evidence is currently available supporting the involvement of these regions in Ca^2+^ inactivation of TRPV5 and TRPV6.

Interestingly, it has recently been reported that the two lobes of CaM bind to two separate subdomains within the distal C-CaMBD of TRPV6 in a 1:2 stoichiometry, with C-lobe constitutively binding to a region formed between S691 and L703 at low Ca^2+^, while high Ca^2+^ triggers the binding of N-lobe to a region between L707 and R708 [79]. A “two-tail” model has therefore been proposed. In accordance with this model, under resting low Ca^2+^, C-lobe CaM constitutively binds to the S691-L703 region through W695 on a C-tail of TRPV6. An elevation in [Ca^2+^]_i_ due to channel opening, loads Ca^2+^ onto N-lobe. Ca^2+^-loaded N-lobe then switches to bind to a second C-tail from another subunit leading to channel inactivation. Indeed, a TRPV6 mutant (L707A) enhanced N-lobe binding and also promoted TRPV6 inactivation [79]. N-lobe is therefore the determinant of channel inactivation. If this is the case, the N-lobe mutant CaM_12_ deficient in Ca^2+^ binding should also abrogate Ca^2+^-dependent inactivation of TRPV6, which remains to be tested. Clearly, more structural and functional evidence will be required to validate this model.

Ca^2+^-induced inactivation of TRPV5 and TRPV6 is also modulated by other signaling pathways that interfere with CaM binding. For example, increased phosphorylation of C-CaMBD in TRPV5 induced by parathyroid hormone impaired CaM binding and therefore enhanced TRPV5 activity [74]. Similarly, phosphorylation of CaM binding region in TRPV6 by PKC also counteracted CaM binding and consequently slowed down channel inactivation [76].

Furthermore, a CaM-independent mechanism has also been suggested to be involved in TRPV6 inactivation [75]. This involves Ca^2+^-induced activation of PLC and subsequent hydrolysis of PIP_2_, similar to that found in TRPV1 and TRPV2 channels. Indeed, PIP_2_ has been found to be sufficient to activate TRPV6, and, moreover, Ca^2+^-evoked inactivation of TRPV6 has found to be reduced by either inhibiting PLC or by dialysis of PIP_2_ into the cell [80,81]. Inactivation of TRPV6 caused by Ca^2+^ is therefore the collective action of both Ca^2+^/CaM and PIP_2_ depletion [82].

## 7. TRPA1

TRPA1 channels detect diverse noxious thermal, chemical and mechanical stimuli, acting as a damage sensor and involved in various disease conditions, such as pain, itch, neuropathy and inflammation [3,10,83]. Ca^2+^ plays a significant role in the regulation of TRPA1. Firstly, Ca^2+^ directly activates TRPA1 and is essential for the basal responses of TRPA1 at lower Ca^2+^ concentrations (<1 mM) [84,85,86]. Ca^2+^ activation of TRPA1 may underlie diversified functions of TRPA1 channels. Indeed, Ca^2+^-dependent activation of TRPA1 has been proposed as a mechanism of TRPA1 activation by noxious cold [84]. Elevations in [Ca^2+^]_i_ by Gq-PLC-coupled receptors also activate TRPA1. TRPA1 may also be potentiated by Ca^2+^ influx through other nearby Ca^2+^-permeable channels, such as TRPV1, that form a complex with TRPA1 [87,88]. Secondly, Ca^2+^ inhibits basal TRPA1 responses and rapidly inactivates TRPA1 at higher Ca^2+^ concentrations (>1 mM) [86,89]. Ca^2+^ therefore exerts a dual and opposite effect on TRPA1 [86].

However, the mechanisms of Ca^2+^ regulation of TRPA1 have been controversial. Previous studies have suggested that the direct binding of Ca^2+^ to TRPA1 is responsible and have excluded the role of CaM in these processes, since dominant-negative CaM_1234_ was found to have no effect in Ca^2+^ regulation of TRPA1 [84,85,90]. On the contrary, we demonstrated that CaM is not only responsible for Ca^2+^-dependent potentiation (CDP) and inactivation (CDI) of TRPA1, but also determines the opposite effects of different Ca^2+^ levels on the basal responses of TRPA1 [86]. Importantly, TRPA1-CaM binding was Ca^2+^-dependent, and Ca^2+^-binding deficient CaM_1234_ did not bind to TRPA1 [86], which explains the lack of effects of CaM_1234_ in the regulation of TRPA1 observed previously [84]. We also identified a non-canonical CaMBD containing 17 amino acids in the C-terminus of TRPA1. Mutations with this region either abolished or diminished CDP and CDI [86]. However, it remains unclear how CaM mediates the opposite Ca^2+^ effects on TRPA1 and whether other regions of TRPA1 are also involved. In particular, the N-terminus of TRPA1 exhibited weak CaM binding and has been shown to be critical for Ca^2+^-induced desensitization of TRPA1 [86,91].

## 8. TRPM2

TRPM2 detects warm temperatures and is implicated in thermal sensation and regulation [92,93]. Notably, TRPM2 is also activated by reactive oxygen species (ROS) and ADP-ribose (ADPR) produced during oxidative stress and ischemia, functioning as a sensor for oxidative stress [9]. Ca^2+^ influx and Ca^2+^ signaling triggered by TRPM2 activation during oxidative stress is critical to many pathological processes such as cytokine production, cell death and immune and inflammatory diseases [9].

In addition to initiating downstream Ca^2+^ signaling, Ca^2+^ also activates TRPM2 and even TRPM2 splice variants that are insensitive to the channel agonist ADPR, suggesting that Ca^2+^ and ADPR open TRPM2 through distinct mechanisms [94]. Interestingly, Ca^2+^ enhanced ADPR-induced activation of TRPM2, but in the absence of Ca^2+^, ADPR was unable to activate TRPM2 [94,95]. Ca^2+^ therefore directly gates and modulates TRPM2 independently of ADPR [94,95,96]. Both Ca^2+^-dependent activation and modulation of TRPM2 are mediated by CaM, based on the following evidence: (1) dominant-negative CaM_1234_ inhibited TRPM2 activation; (2) CaM bound to an IQ-like motif domain in the N-terminus of TRPM2 in a Ca^2+^-dependent manner; (3) mutations in the IQ motif of TRPM2 impaired CaM binding and prevented TRPM2 activation by Ca^2+^ and ADPR [94,96]. Taken together, Ca^2+^ determines the activation and function of TRPM2.

A question remaining unclear is whether Ca^2+^-induced inactivation of TRPM2 [95] is mediated by CaM or by different Ca^2+^-interaction sites yet to be investigated.

## 9. TRPM3

TRPM3 is a sensor for noxious heat critical for heat nociception together with TRPV1 and TRPA1 [97,98]. TRPM3 is permeable to Ca^2+^ and constitutively active [99,100]. Ca^2+^ influx by TRPM3 was shown to be enhanced by depleting intracellular Ca^2+^ stores or by activating the Gq-coupled muscarinic receptor [100], suggesting the Ca^2+^-dependent activation of TRPM3. Interestingly, the N-terminus of TRPM3 contains two regions responsible for interaction with CaM and S100A1, two Ca^2+^-binding proteins [101]. However, neither Ca^2+^-dependent activation of TRPM3 nor the functional role of the postulated CaM binding domains has been directly investigated.

Interestingly, a recent study showed that activation of the Gq-coupled M1 muscarinic receptors rapidly inhibits TRPM3 through released Gβγ [102]. The direct inhibition of TRPM3 by Gβγ has also been reported by two other independent labs [103,104]. Activation of Gq-coupled receptors may therefore produce a mixed effect on TRPM3: an initial rapid inhibition by Gβγ followed by facilitation of activation of TRPM3 by Ca^2+^ due to concomitant activation of the Gq-PLC pathway. This possibility remains to be tested in the future.

## 10. TRPM4 and TRPM5

Both TRPM4 and TRPM5 channels are involved in the transduction of taste stimuli, such as sweet and bitter [105,106]. In contrast to other TRP channels, TRPM4 and TRPM5 are monovalent-selective and do not conduct divalent cations such as Ca^2+^ [107,108,109]. However, [Ca^2+^]_i_ is both necessary and sufficient for activation of both channels [107,109]. As anticipated, TRPM4 and TRPM5 are also activated by the Gq-protein coupled receptors that cause increases in [Ca^2+^]_i_ [106,107,108,109,110,111].

TRPM5 was shown to be activated by [Ca^2+^]_i_ between 0.3 μM and 1 μM, similar to TRPM4, but inhibited at higher [Ca^2+^]_i_ (≥1 μM) [107], which is in contrast to TRPM4, which exhibits no significant inhibition at higher Ca^2+^ levels [109]. Furthermore, activation of TRPM5 by [Ca^2+^]_i_ was rapidly followed by pronounced channel inactivation or desensitization, leading to the distinctive transient activation of the channel [107], which is different from more persistent activation of TRPM4 [109]. The rapid inactivation of TRPM5 was partially reversed by PIP_2_ and was suggested to be Ca^2+^-dependent [110,112], though it was also proposed as a Ca^2+^-independent process [107]. TRPM4 currents undergo a similar rapid channel rundown or desensitization, a process attributed to the hydrolysis of PIP_2_, a positive regulator of TRPM4 [111,113]. As Ca^2+^ entry through other TRP channels (e.g., TRPV1, TRPV2 and TRPV6, see above) has been shown to be sufficient to induce PIP_2_ hydrolysis, it is therefore likely that PIP_2_ hydrolysis, as a consequence of Ca^2+^-triggered activation of PLC, contributes to the desensitization of TRPM4 and TRPM5.

Two other different mechanisms are also involved in the Ca^2+^ sensitivity of TRPM4. The first mechanism involves CaM, similar to other TRP channels. This is evidenced by the finding that Ca^2+^ activation of TRPM4 is markedly decreased by co-expressing the dominant negative CaM_1234_ and that direct application of CaM to excised TRPM4 channels prevents TRPM4 desensitization [114]. In an attempt to identify CaMBD on TRPM4, several CaM binding sites in the N- and C-termini of TRPM4 were found. Consistently, deletion of C-terminal CaM binding sites impaired Ca^2+^ activation of TRPM4 [114]. The second mechanism is mediated by the direct binding of Ca^2+^ to two negatively charged residues (D1049 and E1062) near and in the TRP domain. Neutralization of these residues reduced Ca^2+^ sensitivity [115]. However, a recently resolved TRPV4 structure revealed that the Ca^2+^ binding site is within the intracellular side of the S1–S4 domain [116]. For TRPM5, Ca^2+^ sensitivity is the direct action of Ca^2+^ on TRPM5, independently of CaM, because Ca^2+^ is sufficient to induce TRPM5 activity in excised patches and CaM inhibitors have been shown to have no effect on the Ca^2+^ activation of TRPM5 [108,110]. However, the exact Ca^2+^ binding sites on TRPM5 have not yet been identified.

## 11. TRPM8

TRPM8 channels are activated by cold temperatures (≤30 °C) and cooling compounds, such as menthol, acting as a cold sensor [117] and setting it apart from the warm- or heat-sensitive TRPM2–5 channels [92,97,118].

TRPM8 is permeable to Ca^2+^, and Ca^2+^ influx upon channel opening triggers rapid channel desensitization [119], a process thought to underlie cold adaptation. In the absence of Ca^2+^, TRPM8 currents are substantially larger and lack desensitization [119]. It was thought that Ca^2+^ acts by mainly activating Ca^2+^-sensitive PLCδ4, leading to hydrolysis of PIP_2_, a regulator essential for maintaining channel activity [120,121,122,123]. Consistently, TRPM8 currents were shown to be larger and to exhibit reduced desensitization in PLCδ4-deficient sensory neurons [123]. However, the significant desensitization of TRPM8 was still observed in PLCδ4-lacking neurons [123], suggesting other unknown mechanisms that also underlie Ca^2+^-dependent desensitization of TRPM8. Nevertheless, PLCδ4-lacking mice exhibited increased duration and events of TRPM8-dependent nocifensive behaviors in response to cooling [123]. Similarly, pharmacologically blocking PLC also inhibited the responses of paw withdrawal of the mice to cooling [124]. These experiments suggest that PLC-mediated hydrolysis of PIP_2_ underlies TRPM8 desensitization and cold adaptation in vivo.

## 12. TRPP2

TRPP2 is also known as polycystin-2 (PC2) or PKD2 and is permeable to Ca^2+^ [125]. Mutations in TRPP2 is a cause of autosomal dominant polycystic kidney disease (ADPKD), a consequence thought to be due to aberrant calcium signaling of the mutated channel [126]. Understanding the interaction between TRPP2 and Ca^2+^ is therefore important for elucidating the mechanisms of ADPKD.

Ca^2+^ exerts a dual effect on TRPP2 with lower concentrations of Ca^2+^ (≤0.3 μM) facilitating, and higher concentrations of Ca^2+^ inhibiting, the channel, leading to a bell-shaped Ca^2+^ dependence [127]. The Ca^2+^ sensitivity of TRPP2 channels is believed to be due to a direct binding of Ca^2+^ to the channel. Indeed, a single Ca^2+^ binding site was revealed by NMR in the EF-hand domain (720–797) in the C terminus of TRPP2 [128]. Mutation of the EF-hand domain abolished Ca^2+^ binding and rendered the mutated channel inactive [129,130], suggesting that the EF hand domain is a Ca^2+^ sensor. On the other hand, phosphorylation of TRPP2 at S812 close to the EF-hand domain by casein kinase II (CK2) increased the Ca^2+^ sensitivity of TRPP channels, because mutation of S812 markedly reduced Ca^2+^-dependent activation and inactivation of TRPP2, leading to a positive shift in Ca^2+^-dependence [127]. Presumably, phosphorylation affects the binding affinity of Ca^2+^ to the EF hand domain. However, the single Ca^2+^ binding site in the EF hand domain cannot account for the dual effects of Ca^2+^ on TRPP2. Therefore, Ca^2+^-dependent regulation of TRPP2 likely involves other unknown mechanisms.

## 13. Concluding Remarks

Spatial and temporal control of Ca^2+^ signaling is necessary for many physiological processes. TRP ion channels are not only the major contributors to Ca^2+^ signaling, but also are the targets of Ca^2+^ signaling, ensuring rapid control of functions of TRP ion channels and associated Ca^2+^ signaling. Ca^2+^-regulation of TRP ion channels largely involves three major mechanisms: direct binding of Ca^2+^ to channels, Ca^2+^-sensing CaM and Ca^2+^-dependent hydrolysis of PIP_2_. Of them, CaM is probably the most common mechanism in mediating Ca^2+^-dependent regulation of TRP ion channels. CaM not only directly senses different levels of Ca^2+^ through two different lobes, but also serves as a multifunctional effector protein, providing vast versatility for decoding Ca^2+^ signals and modulating TRP ion channels. Future research is required to further understand diversified regulation of TRP ion channels by CaM. Accumulating evidence also suggests the presence of other Ca^2+^-dependent mechanisms critical in the regulation of TRP ion channels, which remain to be investigated.

## References

[1] Venkatachalam K., Montell C. (2007). TRP channels. Annu. Rev. Biochem..

[2] Nilius B., Owsianik G., Voets T., Peters J.A. (2007). Transient receptor potential cation channels in disease. Physiol. Rev..

[3] Zhang X. (2015). Molecular sensors and modulators of thermoreception. Channels.

[4] Julius D. (2013). TRP channels and pain. Annu. Rev. Cell Dev. Biol..

[5] Nilius B., Owsianik G. (2011). The transient receptor potential family of ion channels. Genome Biol..

[6] Zhang X. (2015). Targeting TRP ion channels for itch relief. N.-S. Arch. Pharmacol..

[7] Shigeto M., Ramracheya R., Tarasov A.I., Cha C.Y., Chibalina M.V., Hastoy B., Philippaert K., Reinbothe T., Rorsman N., Salehi A. (2015). GLP-1 stimulates insulin secretion by PKC-dependent TRPM4 and TRPM5 activation. J. Clin. Investig..

[8] Uchida K., Dezaki K., Damdindorj B., Inada H., Shiuchi T., Mori Y., Yada T., Minokoshi Y., Tominaga M. (2011). Lack of TRPM2 impaired insulin secretion and glucose metabolisms in mice. Diabetes.

[9] Yamamoto S., Shimizu S. (2016). Targeting TRPM2 in ROS-Coupled Diseases. Pharmaceuticals.

[10] Bautista D.M., Pellegrino M., Tsunozaki M. (2013). TRPA1: A gatekeeper for inflammation. Annu. Rev. Physiol..

[11] Tai Y., Yang S., Liu Y., Shao W. (2017). TRPC Channels in Health and Disease. Adv. Exp. Med. Biol..

[12] Venkatachalam K., Wong C.O., Zhu M.X. (2015). The role of TRPMLs in endolysosomal trafficking and function. Cell Calcium.

[13] Chin D., Means A.R. (2000). Calmodulin: A prototypical calcium sensor. Trends Cell Biol..

[14] James P., Vorherr T., Carafoli E. (1995). Calmodulin-binding domains: Just two faced or multi-faceted?. Trends Biochem. Sci..

[15] DeMaria C.D., Soong T.W., Alseikhan B.A., Alvania R.S., Yue D.T. (2001). Calmodulin bifurcates the local Ca^2+^ signal that modulates P/Q-type Ca^2+^ channels. Nature.

[16] Yang T., Hendrickson W.A., Colecraft H.M. (2014). Preassociated apocalmodulin mediates Ca^2+^-dependent sensitization of activation and inactivation of TMEM16A/16B Ca^2+^-gated Cl^−^ channels. Proc. Natl. Acad. Sci. USA.

[17] Hoeflich K.P., Ikura M. (2002). Calmodulin in action: Diversity in target recognition and activation mechanisms. Cell.

[18] Xia X.M., Fakler B., Rivard A., Wayman G., Johnson-Pais T., Keen J.E., Ishii T., Hirschberg B., Bond C.T., Lutsenko S. (1998). Mechanism of calcium gating in small-conductance calcium-activated potassium channels. Nature.

[19] Schumacher M.A., Rivard A.F., Bachinger H.P., Adelman J.P. (2001). Structure of the gating domain of a Ca^2+^-activated K^+^ channel complexed with Ca^2+^/calmodulin. Nature.

[20] Ben-Johny M., Yang P.S., Niu J., Yang W., Joshi-Mukherjee R., Yue D.T. (2014). Conservation of Ca^2+^/Calmodulin Regulation across Na and Ca^2+^ Channels. Cell.

[21] Ben-Johny M., Dick I.E., Sang L., Limpitikul W.B., Kang P.W., Niu J., Banerjee R., Yang W., Babich J.S., Issa J.B. (2015). Towards a Unified Theory of Calmodulin Regulation (Calmodulation) of Voltage-Gated Calcium and Sodium Channels. Curr. Mol. Pharmacol..

[22] Zhu M.X. (2005). Multiple roles of calmodulin and other Ca^2+^-binding proteins in the functional regulation of TRP channels. Pflügers Arch..

[23] Caterina M.J., Leffler A., Malmberg A.B., Martin W.J., Trafton J., Petersen-Zeitz K.R., Koltzenburg M., Basbaum A.I., Julius D. (2000). Impaired nociception and pain sensation in mice lacking the capsaicin receptor. Science.

[24] Davis J.B., Gray J., Gunthorpe M.J., Hatcher J.P., Davey P.T., Overend P., Harries M.H., Latcham J., Clapham C., Atkinson K. (2000). Vanilloid receptor-1 is essential for inflammatory thermal hyperalgesia. Nature.

[25] Caterina M.J., Schumacher M.A., Tominaga M., Rosen T.A., Levine J.D., Julius D. (1997). The capsaicin receptor: A heat-activated ion channel in the pain pathway. Nature.

[26] Koplas P.A., Rosenberg R.L., Oxford G.S. (1997). The role of calcium in the desensitization of capsaicin responses in rat dorsal root ganglion neurons. J. Neurosci..

[27] Numazaki M., Tominaga T., Takeuchi K., Murayama N., Toyooka H., Tominaga M. (2003). Structural determinant of TRPV1 desensitization interacts with calmodulin. Proc. Natl. Acad. Sci. USA.

[28] Rosenbaum T., Gordon-Shaag A., Munari M., Gordon S.E. (2004). Ca^2+^/calmodulin modulates TRPV1 activation by capsaicin. J. Gen. Physiol..

[29] Lishko P.V., Procko E., Jin X., Phelps C.B., Gaudet R. (2007). The ankyrin repeats of TRPV1 bind multiple ligands and modulate channel sensitivity. Neuron.

[30] Lau S.Y., Procko E., Gaudet R. (2012). Distinct properties of Ca^2+^-calmodulin binding to N- and C-terminal regulatory regions of the TRPV1 channel. J. Gen. Physiol..

[31] Grycova L., Lansky Z., Friedlova E., Obsilova V., Janouskova H., Obsil T., Teisinger J. (2008). Ionic interactions are essential for TRPV1 C-terminus binding to calmodulin. Biochem. Biophys. Res. Commun..

[32] Kwak J., Wang M.H., Hwang S.W., Kim T.Y., Lee S.Y., Oh U. (2000). Intracellular ATP increases capsaicin-activated channel activity by interacting with nucleotide-binding domains. J. Neurosci..

[33] Liu B., Zhang C., Qin F. (2005). Functional recovery from desensitization of vanilloid receptor TRPV1 requires resynthesis of phosphatidylinositol 4,5-bisphosphate. J. Neurosci..

[34] Liao M., Cao E., Julius D., Cheng Y. (2013). Structure of the TRPV1 ion channel determined by electron cryo-microscopy. Nature.

[35] Docherty R.J., Yeats J.C., Bevan S., Boddeke H.W. (1996). Inhibition of calcineurin inhibits the desensitization of capsaicin-evoked currents in cultured dorsal root ganglion neurones from adult rats. Pflugers Arch..

[36] Mohapatra D.P., Nau C. (2005). Regulation of Ca^2+^-dependent desensitization in the vanilloid receptor TRPV1 by calcineurin and cAMP-dependent protein kinase. J. Biol. Chem..

[37] Zhang X., Li L., McNaughton P.A. (2008). Proinflammatory mediators modulate the heat-activated ion channel TRPV1 via the scaffolding protein AKAP79/150. Neuron.

[38] Por E.D., Samelson B.K., Belugin S., Akopian A.N., Scott J.D., Jeske N.A. (2010). PP2B/calcineurin-mediated desensitization of TRPV1 does not require AKAP150. Biochem. J..

[39] Bhave G., Zhu W., Wang H., Brasier D.J., Oxford G.S., Gereau R.W. (2002). cAMP-dependent protein kinase regulates desensitization of the capsaicin receptor (VR1) by direct phosphorylation. Neuron.

[40] Bhave G., Hu H.J., Glauner K.S., Zhu W., Wang H., Brasier D.J., Oxford G.S., Gereau R.W. (2003). Protein kinase C phosphorylation sensitizes but does not activate the capsaicin receptor transient receptor potential vanilloid 1 (TRPV1). Proc. Natl. Acad. Sci. USA.

[41] Li L., Hasan R., Zhang X. (2014). The Basal Thermal Sensitivity of the TRPV1 Ion Channel Is Determined by PKCbetaII. J. Neurosci..

[42] Mohapatra D.P., Nau C. (2003). Desensitization of capsaicin-activated currents in the vanilloid receptor TRPV1 is decreased by the cyclic AMP-dependent protein kinase pathway. J. Biol. Chem..

[43] Mandadi S., Numazaki M., Tominaga M., Bhat M.B., Armati P.J., Roufogalis B.D. (2004). Activation of protein kinase C reverses capsaicin-induced calcium-dependent desensitization of TRPV1 ion channels. Cell Calcium.

[44] Mandadi S., Tominaga T., Numazaki M., Murayama N., Saito N., Armati P.J., Roufogalis B.D., Tominaga M. (2006). Increased sensitivity of desensitized TRPV1 by PMA occurs through PKCepsilon-mediated phosphorylation at S800. Pain.

[45] Stein A.T., Ufret-Vincenty C.A., Hua L., Santana L.F., Gordon S.E. (2006). Phosphoinositide 3-kinase binds to TRPV1 and mediates NGF-stimulated TRPV1 trafficking to the plasma membrane. J. Gen. Physiol..

[46] Lukacs V., Thyagarajan B., Varnai P., Balla A., Balla T., Rohacs T. (2007). Dual regulation of TRPV1 by phosphoinositides. J. Neurosci..

[47] Poblete H., Oyarzun I., Olivero P., Comer J., Zuniga M., Sepulveda R.V., Baez-Nieto D., Gonzalez Leon C., Gonzalez-Nilo F., Latorre R. (2015). Molecular determinants of phosphatidylinositol 4,5-bisphosphate (PI(4,5)P2) binding to transient receptor potential V1 (TRPV1) channels. J. Biol. Chem..

[48] Lukacs V., Yudin Y., Hammond G.R., Sharma E., Fukami K., Rohacs T. (2013). Distinctive changes in plasma membrane phosphoinositides underlie differential regulation of TRPV1 in nociceptive neurons. J. Neurosci..

[49] Caterina M.J., Rosen T.A., Tominaga M., Brake A.J., Julius D. (1999). A capsaicin-receptor homologue with a high threshold for noxious heat. Nature.

[50] Park U., Vastani N., Guan Y., Raja S.N., Koltzenburg M., Caterina M.J. (2011). TRP vanilloid 2 knock-out mice are susceptible to perinatal lethality but display normal thermal and mechanical nociception. J. Neurosci..

[51] Link T.M., Park U., Vonakis B.M., Raben D.M., Soloski M.J., Caterina M.J. (2010). TRPV2 has a pivotal role in macrophage particle binding and phagocytosis. Nat. Immunol..

[52] Peralvarez-Marin A., Donate-Macian P., Gaudet R. (2013). What do we know about the transient receptor potential vanilloid 2 (TRPV2) ion channel?. FEBS J..

[53] Mercado J., Gordon-Shaag A., Zagotta W.N., Gordon S.E. (2010). Ca^2+^-dependent desensitization of TRPV2 channels is mediated by hydrolysis of phosphatidylinositol 4,5-bisphosphate. J. Neurosci..

[54] Phelps C.B., Wang R.R., Choo S.S., Gaudet R. (2010). Differential regulation of TRPV1, TRPV3, and TRPV4 sensitivity through a conserved binding site on the ankyrin repeat domain. J. Biol. Chem..

[55] Holakovska B., Grycova L., Bily J., Teisinger J. (2011). Characterization of calmodulin binding domains in TRPV2 and TRPV5 C-tails. Amino Acids.

[56] Nilius B., Biro T., Owsianik G. (2013). TRPV3: Time to decipher a poorly understood family member!. J. Physiol..

[57] Huang S.M., Li X., Yu Y., Wang J., Caterina M.J. (2011). TRPV3 and TRPV4 ion channels are not major contributors to mouse heat sensation. Mol. Pain.

[58] Moqrich A., Hwang S.W., Earley T.J., Petrus M.J., Murray A.N., Spencer K.S., Andahazy M., Story G.M., Patapoutian A. (2005). Impaired thermosensation in mice lacking TRPV3, a heat and camphor sensor in the skin. Science.

[59] Marics I., Malapert P., Reynders A., Gaillard S., Moqrich A. (2014). Acute heat-evoked temperature sensation is impaired but not abolished in mice lacking TRPV1 and TRPV3 channels. PLoS ONE.

[60] Xiao R., Tang J., Wang C., Colton C.K., Tian J., Zhu M.X. (2008). Calcium plays a central role in the sensitization of TRPV3 channel to repetitive stimulations. J. Biol. Chem..

[61] Xu H., Delling M., Jun J.C., Clapham D.E. (2006). Oregano, thyme and clove-derived flavors and skin sensitizers activate specific TRP channels. Nat. Neurosci..

[62] Moore C., Gupta R., Jordt S.E., Chen Y., Liedtke W.B. (2017). Regulation of Pain and Itch by TRP Channels. Neurosci. Bull..

[63] Verma P., Kumar A., Goswami C. (2010). TRPV4-mediated channelopathies. Channels.

[64] Watanabe H., Vriens J., Janssens A., Wondergem R., Droogmans G., Nilius B. (2003). Modulation of TRPV4 gating by intra- and extracellular Ca^2+^. Cell Calcium.

[65] Strotmann R., Schultz G., Plant T.D. (2003). Ca^2+^-dependent potentiation of the nonselective cation channel TRPV4 is mediated by a C-terminal calmodulin binding site. J. Biol. Chem..

[66] Strotmann R., Semtner M., Kepura F., Plant T.D., Schoneberg T. (2010). Interdomain interactions control Ca^2+^-dependent potentiation in the cation channel TRPV4. PLoS ONE.

[67] Loukin S.H., Teng J., Kung C. (2015). A channelopathy mechanism revealed by direct calmodulin activation of TrpV4. Proc. Natl. Acad. Sci. USA.

[68] Garcia-Elias A., Lorenzo I.M., Vicente R., Valverde M.A. (2008). IP3 receptor binds to and sensitizes TRPV4 channel to osmotic stimuli via a calmodulin-binding site. J. Biol. Chem..

[69] Takahashi N., Hamada-Nakahara S., Itoh Y., Takemura K., Shimada A., Ueda Y., Kitamata M., Matsuoka R., Hanawa-Suetsugu K., Senju Y. (2014). TRPV4 channel activity is modulated by direct interaction of the ankyrin domain to PI(4,5)P2. Nat. Commun..

[70] Nijenhuis T., Hoenderop J.G., Bindels R.J. (2005). TRPV5 and TRPV6 in Ca^2+^ (re)absorption: Regulating Ca^2+^ entry at the gate. Pflugers Arch..

[71] Saotome K., Singh A.K., Yelshanskaya M.V., Sobolevsky A.I. (2016). Crystal structure of the epithelial calcium channel TRPV6. Nature.

[72] Nilius B., Prenen J., Hoenderop J.G., Vennekens R., Hoefs S., Weidema A.F., Droogmans G., Bindels R.J. (2002). Fast and slow inactivation kinetics of the Ca^2+^ channels ECaC1 and ECaC2 (TRPV5 and TRPV6). Role of the intracellular loop located between transmembrane segments 2 and 3. J. Biol. Chem..

[73] Nilius B., Weidema F., Prenen J., Hoenderop J.G., Vennekens R., Hoefs S., Droogmans G., Bindels R.J. (2003). The carboxyl terminus of the epithelial Ca^2+^ channel ECaC1 is involved in Ca^2+^-dependent inactivation. Pflugers Arch..

[74] De Groot T., Kovalevskaya N.V., Verkaart S., Schilderink N., Felici M., van der Hagen E.A., Bindels R.J., Vuister G.W., Hoenderop J.G. (2011). Molecular mechanisms of calmodulin action on TRPV5 and modulation by parathyroid hormone. Mol. Cell. Biol..

[75] Derler I., Hofbauer M., Kahr H., Fritsch R., Muik M., Kepplinger K., Hack M.E., Moritz S., Schindl R., Groschner K. (2006). Dynamic but not constitutive association of calmodulin with rat TRPV6 channels enables fine tuning of Ca^2+^-dependent inactivation. J. Physiol..

[76] Niemeyer B.A., Bergs C., Wissenbach U., Flockerzi V., Trost C. (2001). Competitive regulation of CaT-like-mediated Ca^2+^ entry by protein kinase C and calmodulin. Proc. Natl. Acad. Sci. USA.

[77] Lambers T.T., Weidema A.F., Nilius B., Hoenderop J.G., Bindels R.J. (2004). Regulation of the mouse epithelial Ca^2+^ channel TRPV6 by the Ca^2+^-sensor calmodulin. J. Biol. Chem..

[78] Kovalevskaya N.V., Bokhovchuk F.M., Vuister G.W. (2012). The TRPV5/6 calcium channels contain multiple calmodulin binding sites with differential binding properties. J. Struct. Funct. Genom..

[79] Bate N., Caves R.E., Skinner S.P., Goult B.T., Basran J., Mitcheson J.S., Vuister G.W. (2018). A Novel Mechanism for Calmodulin Dependent Inactivation of Transient Receptor Potential Vanilloid 6. Biochemistry.

[80] Thyagarajan B., Lukacs V., Rohacs T. (2008). Hydrolysis of Phosphatidylinositol 4,5-Bisphosphate Mediates Calcium-Induced Inactivation of TRPV6 Channels. J. Biol. Chem..

[81] Thyagarajan B., Benn B.S., Christakos S., Rohacs T. (2009). Phospholipase C-mediated regulation of transient receptor potential vanilloid 6 channels: Implications in active intestinal Ca^2+^ transport. Mol. Pharmacol..

[82] Cao C., Zakharian E., Borbiro I., Rohacs T. (2013). Interplay between calmodulin and phosphatidylinositol 4,5-bisphosphate in Ca^2+^-induced inactivation of transient receptor potential vanilloid 6 channels. J. Biol. Chem..

[83] Zygmunt P.M., Hogestatt E.D. (2014). TRPA1. Handb. Exp. Pharmacol..

[84] Zurborg S., Yurgionas B., Jira J.A., Caspani O., Heppenstall P.A. (2007). Direct activation of the ion channel TRPA1 by Ca^2+^. Nat. Neurosci..

[85] Doerner J.F., Gisselmann G., Hatt H., Wetzel C.H. (2007). Transient receptor potential channel A1 is directly gated by calcium ions. J. Biol. Chem..

[86] Hasan R., Leeson-Payne A.T., Jaggar J.H., Zhang X. (2017). Calmodulin is responsible for Ca^2+^-dependent regulation of TRPA1 Channels. Sci. Rep..

[87] Weng H.J., Patel K.N., Jeske N.A., Bierbower S.M., Zou W., Tiwari V., Zheng Q., Tang Z., Mo G.C., Wang Y. (2015). Tmem100 Is a Regulator of TRPA1-TRPV1 Complex and Contributes to Persistent Pain. Neuron.

[88] Bautista D.M., Jordt S.E., Nikai T., Tsuruda P.R., Read A.J., Poblete J., Yamoah E.N., Basbaum A.I., Julius D. (2006). TRPA1 mediates the inflammatory actions of environmental irritants and proalgesic agents. Cell.

[89] Wang Y.Y., Chang R.B., Waters H.N., McKemy D.D., Liman E.R. (2008). The nociceptor ion channel TRPA1 is potentiated and inactivated by permeating calcium ions. J. Biol. Chem..

[90] Sura L., Zima V., Marsakova L., Hynkova A., Barvik I., Vlachova V. (2012). C-terminal acidic cluster is involved in Ca^2+^-induced regulation of human transient receptor potential ankyrin 1 channel. J. Biol. Chem..

[91] Cordero-Morales J.F., Gracheva E.O., Julius D. (2011). Cytoplasmic ankyrin repeats of transient receptor potential A1 (TRPA1) dictate sensitivity to thermal and chemical stimuli. Proc. Natl. Acad. Sci. USA.

[92] Tan C.H., McNaughton P.A. (2016). The TRPM2 ion channel is required for sensitivity to warmth. Nature.

[93] Song K., Wang H., Kamm G.B., Pohle J., Reis F.C., Heppenstall P., Wende H., Siemens J. (2016). The TRPM2 channel is a hypothalamic heat sensor that limits fever and can drive hypothermia. Science.

[94] Du J., Xie J., Yue L. (2009). Intracellular calcium activates TRPM2 and its alternative spliced isoforms. Proc. Natl. Acad. Sci. USA.

[95] Starkus J., Beck A., Fleig A., Penner R. (2007). Regulation of TRPM2 by extra- and intracellular calcium. J. Gen. Physiol..

[96] Tong Q., Zhang W., Conrad K., Mostoller K., Cheung J.Y., Peterson B.Z., Miller B.A. (2006). Regulation of the transient receptor potential channel TRPM2 by the Ca^2+^ sensor calmodulin. J. Biol. Chem..

[97] Vriens J., Owsianik G., Hofmann T., Philipp S.E., Stab J., Chen X., Benoit M., Xue F., Janssens A., Kerselaers S. (2011). TRPM3 is a nociceptor channel involved in the detection of noxious heat. Neuron.

[98] Vandewauw I., de Clercq K., Mulier M., Held K., Pinto S., van Ranst N., Segal A., Voet T., Vennekens R., Zimmermann K. (2018). A TRP channel trio mediates acute noxious heat sensing. Nature.

[99] Grimm C., Kraft R., Sauerbruch S., Schultz G., Harteneck C. (2003). Molecular and functional characterization of the melastatin-related cation channel TRPM3. J. Biol. Chem..

[100] Lee N., Chen J., Sun L., Wu S., Gray K.R., Rich A., Huang M., Lin J.H., Feder J.N., Janovitz E.B. (2003). Expression and characterization of human transient receptor potential melastatin 3 (hTRPM3). J. Biol. Chem..

[101] Holakovska B., Grycova L., Jirku M., Sulc M., Bumba L., Teisinger J. (2012). Calmodulin and S100A1 protein interact with N terminus of TRPM3 channel. J. Biol. Chem..

[102] Badheka D., Yudin Y., Borbiro I., Hartle C.M., Yazici A., Mirshahi T., Rohacs T. (2017). Inhibition of Transient Receptor Potential Melastatin 3 ion channels by G-protein betagamma subunits. Elife.

[103] Dembla S., Behrendt M., Mohr F., Goecke C., Sondermann J., Schneider F.M., Schmidt M., Stab J., Enzeroth R., Leitner M.G. (2017). Anti-nociceptive action of peripheral mu-opioid receptors by G-beta-gamma protein-mediated inhibition of TRPM3 channels. Elife.

[104] Quallo T., Alkhatib O., Gentry C., Andersson D.A., Bevan S. (2017). G protein betagamma subunits inhibit TRPM3 ion channels in sensory neurons. Elife.

[105] Dutta Banik D., Martin L.E., Freichel M., Torregrossa A.M., Medler K.F. (2018). TRPM4 and TRPM5 are both required for normal signaling in taste receptor cells. Proc. Natl. Acad. Sci. USA.

[106] Zhang Y., Hoon M.A., Chandrashekar J., Mueller K.L., Cook B., Wu D., Zuker C.S., Ryba N.J. (2003). Coding of sweet, bitter, and umami tastes: Different receptor cells sharing similar signaling pathways. Cell.

[107] Prawitt D., Monteilh-Zoller M.K., Brixel L., Spangenberg C., Zabel B., Fleig A., Penner R. (2003). TRPM5 is a transient Ca^2+^-activated cation channel responding to rapid changes in [Ca^2+^]_i_. Proc. Natl. Acad. Sci. USA.

[108] Hofmann T., Chubanov V., Gudermann T., Montell C. (2003). TRPM5 is a voltage-modulated and Ca^2+^-activated monovalent selective cation channel. Curr. Biol..

[109] Launay P., Fleig A., Perraud A.L., Scharenberg A.M., Penner R., Kinet J.P. (2002). TRPM4 is a Ca^2+^-activated nonselective cation channel mediating cell membrane depolarization. Cell.

[110] Liu D., Liman E.R. (2003). Intracellular Ca^2+^ and the phospholipid PIP_2_ regulate the taste transduction ion channel TRPM5. Proc. Natl. Acad. Sci. USA.

[111] Nilius B., Mahieu F., Prenen J., Janssens A., Owsianik G., Vennekens R., Voets T. (2006). The Ca^2+^-activated cation channel TRPM4 is regulated by phosphatidylinositol 4,5-biphosphate. EMBO J..

[112] Zhang Z., Zhao Z., Margolskee R., Liman E. (2007). The transduction channel TRPM5 is gated by intracellular calcium in taste cells. J. Neurosci..

[113] Zhang Z., Okawa H., Wang Y., Liman E.R. (2005). Phosphatidylinositol 4,5-bisphosphate rescues TRPM4 channels from desensitization. J. Biol. Chem..

[114] Nilius B., Prenen J., Tang J., Wang C., Owsianik G., Janssens A., Voets T., Zhu M.X. (2005). Regulation of the Ca^2+^ sensitivity of the nonselective cation channel TRPM4. J. Biol. Chem..

[115] Yamaguchi S., Tanimoto A., Otsuguro K., Hibino H., Ito S. (2014). Negatively charged amino acids near and in transient receptor potential (TRP) domain of TRPM4 channel are one determinant of its Ca^2+^ sensitivity. J. Biol. Chem..

[116] Autzen H.E., Myasnikov A.G., Campbell M.G., Asarnow D., Julius D., Cheng Y. (2018). Structure of the human TRPM4 ion channel in a lipid nanodisc. Science.

[117] Bautista D.M., Siemens J., Glazer J.M., Tsuruda P.R., Basbaum A.I., Stucky C.L., Jordt S.E., Julius D. (2007). The menthol receptor TRPM8 is the principal detector of environmental cold. Nature.

[118] Talavera K., Yasumatsu K., Voets T., Droogmans G., Shigemura N., Ninomiya Y., Margolskee R.F., Nilius B. (2005). Heat activation of TRPM5 underlies thermal sensitivity of sweet taste. Nature.

[119] McKemy D.D., Neuhausser W.M., Julius D. (2002). Identification of a cold receptor reveals a general role for TRP channels in thermosensation. Nature.

[120] Rohacs T., Lopes C.M., Michailidis I., Logothetis D.E. (2005). PI(4,5)P2 regulates the activation and desensitization of TRPM8 channels through the TRP domain. Nat. Neurosci..

[121] Yudin Y., Lukacs V., Cao C., Rohacs T. (2011). Decrease in phosphatidylinositol 4,5-bisphosphate levels mediates desensitization of the cold sensor TRPM8 channels. J. Physiol..

[122] Daniels R.L., Takashima Y., McKemy D.D. (2009). Activity of the neuronal cold sensor TRPM8 is regulated by phospholipase C via the phospholipid phosphoinositol 4,5-bisphosphate. J. Biol. Chem..

[123] Yudin Y., Lutz B., Tao Y.X., Rohacs T. (2016). Phospholipase C delta4 regulates cold sensitivity in mice. J. Physiol..

[124] Brenner D.S., Golden J.P., Vogt S.K., Dhaka A., Story G.M., Gereau R.I. (2014). A dynamic set point for thermal adaptation requires phospholipase C-mediated regulation of TRPM8 in vivo. Pain.

[125] Koulen P., Cai Y., Geng L., Maeda Y., Nishimura S., Witzgall R., Ehrlich B.E., Somlo S. (2002). Polycystin-2 is an intracellular calcium release channel. Nat. Cell Biol..

[126] Yang Y., Ehrlich B.E. (2016). Structural studies of the C-terminal tail of polycystin-2 (PC2) reveal insights into the mechanisms used for the functional regulation of PC2. J. Physiol..

[127] Cai Y., Anyatonwu G., Okuhara D., Lee K.B., Yu Z., Onoe T., Mei C.L., Qian Q., Geng L., Wiztgall R. (2004). Calcium dependence of polycystin-2 channel activity is modulated by phosphorylation at Ser812. J. Biol. Chem..

[128] Petri E.T., Ćelić A., Kennedy S.D., Ehrlich B.E., Boggon T.J., Hodsdon M.E. (2010). Structure of the EF-hand domain of polycystin-2 suggests a mechanism for Ca^2+^-dependent regulation of polycystin-2 channel activity. Proc. Natl. Acad. Sci. USA.

[129] Celic A.S., Petri E.T., Benbow J., Hodsdon M.E., Ehrlich B.E., Boggon T.J. (2012). Calcium-induced conformational changes in C-terminal tail of polycystin-2 are necessary for channel gating. J. Biol. Chem..

[130] Celic A., Petri E.T., Demeler B., Ehrlich B.E., Boggon T.J. (2008). Domain mapping of the polycystin-2 C-terminal tail using de novo molecular modeling and biophysical analysis. J. Biol. Chem..

